# Application of Liquid‐Based Cytology Using the ThinPrep System to Thyroid Fine‐Needle Aspiration Samples

**DOI:** 10.1002/cpz1.70484

**Published:** 2026-10-07

**Authors:** Vargas‐Castellanos Elizabeth, Arenas Avila Lina Alejandra, Rocha Franky

**Affiliations:** ^1^ Hospital Universitario Mayor ‐ Méderi Universidad del Rosario Bogotá Colombia

**Keywords:** cell block preparation, cytopathology, liquid‐based cytology, ThinPrep, thyroid fine‐needle aspiration

## Abstract

Fine‐needle aspiration (FNA) is the gold‐standard diagnostic procedure for the initial evaluation of thyroid nodules because it is minimally invasive, cost‐effective, and highly accurate for distinguishing benign from malignant lesions. However, conventional smear preparation is frequently affected by operator‐dependent variability, uneven cell distribution, blood contamination, and air‐drying artifacts, all of which may compromise specimen adequacy and diagnostic performance. Liquid‐based cytology (LBC) has emerged as an effective alternative, offering standardized specimen processing, improved cellular preservation, and enhanced slide quality. Among the available LBC platforms, the ThinPrep system is one of the most extensively validated technologies for thyroid cytopathology and is increasingly integrated into routine clinical practice and molecular diagnostic workflows. This protocol provides a comprehensive, standardized workflow for processing thyroid FNA specimens using the ThinPrep liquid‐based cytology system. The protocol describes specimen collection, transportation, fixation, processing with the ThinPrep 5000 processor, slide preparation, staining, and quality control procedures to ensure optimal cytomorphological evaluation. Recommendations for acceptable specimen preservation, including transport in PreservCyt solution or alternative fixation methods, are also provided. In addition, the protocol includes procedures for recovering the residual cellular material preserved in the ThinPrep vial for paraffin block processing, which allows for complementary studies, e.g., immunohistochemistry, fluorescence in situ hybridization (FISH), DNA and RNA extraction, and next‐generation sequencing (NGS), thus expanding the diagnostic utility of thyroid cytology beyond conventional microscopic examination. When properly executed, this protocol generates high‐quality monolayer cytology preparations suitable for standardized interpretation according to The Bethesda System for Reporting Thyroid Cytopathology (TBSRTC) while preserving residual material for downstream molecular analyses. Compared with conventional smear cytology, the ThinPrep system improves specimen reproducibility, reduces background artifacts and inadequate samples, facilitates laboratory standardization, and supports precision medicine applications. This protocol provides a practical and reproducible approach for both clinical diagnostic laboratories and translational research. © 2026 The Author(s). *Current Protocols* published by Wiley Periodicals LLC.

**Basic Protocol**: Simultaneous preparation of ThinPrep cytology slides and cell blocks from thyroid fine‐needle aspiration specimens using liquid‐based cytology

**Alternate Protocol**: Alternative ThinPrep FNA processing using CytoLyt and PreservCyt

## INTRODUCTION

Thyroid nodules are among the most common endocrine disorders worldwide, with a prevalence ranging from 34% to 66% depending on the population studied and the imaging modality used (Durante et al., 2018; Uppal et al., 2023). Although most thyroid nodules are benign, 5% to 15% harbor malignancy, making accurate diagnostic evaluation essential for appropriate patient management (Haugen et al., 2016). Fine‐needle aspiration (FNA) cytology is currently considered the gold standard initial diagnostic procedure for thyroid nodule assessment because it is minimally invasive, cost‐effective, and highly sensitive for identifying lesions that require surgical intervention (Park et al., 2023). The implementation of The Bethesda System for Reporting Thyroid Cytopathology (TBSRTC) has further standardized cytological interpretation and improved communication between pathologists and clinicians (Ali et al., 2023).

Conventional smear‐based cytology has historically been the standard method for processing thyroid FNA specimens. However, this approach is associated with several limitations, including uneven cell distribution, air‐drying artifacts, blood contamination, thick cellular aggregates, and operator‐dependent variability in specimen preparation (Erdoğan et al., 2023). These factors may negatively affect diagnostic accuracy and contribute to higher rates of unsatisfactory or nondiagnostic samples. Consequently, liquid‐based cytology (LBC) techniques have emerged as valuable alternatives for improving specimen quality and reproducibility (Wang et al., 2024).

LBC was initially developed for cervical cancer screening and subsequently adapted for non‐gynecological specimens, including thyroid FNA samples (Nasuti et al., 2001). Among the available technologies, the ThinPrep system is one of the most widely adopted and extensively validated platforms. In this method, aspirated cells are suspended in a preservative solution and processed through automated filtration technology to generate a thin, uniform monolayer of cells on a microscope slide (Tulecke & Wang, 2004). This approach minimizes obscuring background elements, improves cellular preservation, and facilitates standardized cytomorphological evaluation (Sauter et al., 2019).

The evolution of liquid‐based cytology has expanded the diagnostic utility of thyroid cytology beyond conventional morphological assessment. Residual material preserved in the collection vial can be used for ancillary studies, including immunohistochemistry, fluorescence in situ hybridization (FISH), and molecular analyses, e.g., DNA and RNA extraction, mutation testing, and next‐generation sequencing (NGS) (da Cunha Santos et al., 2018). These applications are particularly relevant in the management of indeterminate thyroid nodules, where molecular profiling can improve risk stratification and guide therapeutic decision‐making (Fazeli et al., 2020).

Data obtained from ThinPrep‐processed thyroid FNA specimens include cytomorphological features, such as cellularity, architectural arrangements, colloid content, inflammatory background, nuclear atypia, and features associated with benign, indeterminate, or malignant lesions. In addition, preserved residual material may provide genomic, transcriptomic, and proteomic data suitable for translational research and precision oncology applications (Roth et al., 2018).

Compared with conventional smear cytology, the ThinPrep system offers several advantages, including improved specimen standardization, enhanced cellular preservation, reduced obscuring artifacts, decreased rates of inadequate samples, and compatibility with ancillary molecular testing (Steward et al., 2019; Uppal et al., 2023). However, the technique also presents limitations: certain architectural patterns may be altered during processing, some cytological features may appear different from those observed in conventional preparations, and implementation requires dedicated equipment and additional laboratory costs (Tulecke & Wang, 2004). Therefore, laboratories should carefully evaluate workflow requirements, available resources, and intended downstream applications when selecting an appropriate processing method.

This article describes a standardized protocol for processing thyroid fine‐needle aspiration specimens using the ThinPrep liquid‐based cytology system. The Basic Protocol details specimen collection, fixation, processing, slide preparation, staining, and quality control procedures. An Alternate Protocol describes the recovery of residual cellular material for molecular applications, including nucleic acid extraction and downstream genomic analyses.

## STRATEGIC PLANNING

Verify specimen preservation conditions as follows:

Preferred: Specimen received in a ThinPrep vial containing 20 ml PreservCyt solution.
Alternative: Specimen transported under cold‐chain conditions (2° to 8°C) or fixed in 96% ethanol (see recipe) at a 1:1 ratio (fixative).

*NOTE*: Proper fixation is essential to maintain cellular morphology and ensure satisfactory cytological evaluation.

*NOTE*: All protocols involving animals must be reviewed and approved by the appropriate Animal Care and Use Committee and must follow regulations for the care and use of laboratory animals. Appropriate informed consent is necessary for obtaining and use of human study material.

## SIMULTANEOUS PREPARATION OF ThinPrep CYTOLOGY SLIDES AND CELL BLOCKS FROM THYROID FINE‐NEEDLE ASPIRATION SPECIMENS USING LIQUID‐BASED CYTOLOGY

This protocol describes the standardized processing of thyroid FNA specimens using the ThinPrep liquid‐based cytology system while simultaneously preserving material for cell block preparation and ancillary studies. After specimen collection and fixation in PreservCyt solution, the sample is divided into two aliquots: one for conventional ThinPrep slide preparation and the other for cell block generation. Sequential centrifugation and specimen processing with CytoLyt solution and glacial acetic acid reduce blood contamination, mucus, and proteinaceous debris, thereby improving cellular preservation and slide quality. The resulting cell block provides additional material suitable for histopathological evaluation, immunohistochemistry, and molecular testing. When performed correctly, this protocol produces well‐preserved monolayer cytology slides with minimal background interference and an adequate cellular clot for ancillary diagnostic analyses, maximizing the diagnostic yield from a single thyroid FNA specimen.

### Materials

PreservCyt solution (see recipe)
96% ethanol (alternative fixative) (see recipe)
Human plasma solution (see recipe)
Innovin reagent (thromboplastin reagent) (see recipe)
Glacial acetic acid (see recipe)
CytoLyt solution (see recipe)

25‐ to 27‐gauge needle
15‐ml conical centrifuge tube
Centrifuge capable of 725 × *g*
ThinPrep 5000 Processor (Hologic)
Glass microscope slides

*CAUTION*: Glacial acetic acid is highly corrosive. Handle with appropriate personal protective equipment PPE and inside a chemical hood.

#### Specimen collection and preservation

1 Perform ultrasound‐guided fine‐needle aspiration (FNA) using a 25‐ to 27‐gauge needle in accordance with institutional clinical practice.
2 After each needle pass, expel the aspirated material directly into a vial containing 20 ml of PreservCyt solution. Alternatively, if PreservCyt Solution is not used, the aspirated specimen may be fixed in 96% ethanol at a 1:1 specimen‐to‐fixative ratio.
3 Rinse the needle and syringe several times with the preservative solution to maximize cellular recovery.
4 Cap the vial securely and gently invert the vial 8 to 10 times to homogenize the cellular suspension.
5 Label the specimen with two patient identifiers and transport it to the cytopathology laboratory.
6 Store the specimen at room temperature until processing. Process specimens as soon as possible after collection to ensure optimal cellular preservation and minimize cellular degradation.
  Immediate immersion in PreservCyt minimizes air‐drying artifacts, preserves nuclear morphology, reduces cellular degeneration, and maintains residual material suitable for ancillary testing.

#### Specimen aliquoting

If ancillary studies are anticipated, divide the preserved specimen into two aliquots after gentle mixing.

7 Gently invert the PreservCyt vial 8 to 10 times.
8 Transfer approximately half of the specimen into a sterile 15‐ml conical centrifuge tube for cell block preparation.
9 Retain the remaining specimen for ThinPrep cytology processing.
10 Store any residual specimen in the original PreservCyt vial for possible repeat preparation or molecular testing, in accordance with institutional policy.
  Expected outcome: Two representative aliquots suitable for both cytological evaluation and ancillary studies.

#### Initial centrifugation

11 Centrifuge both aliquots for 5 min at 725 × *g*, room temperature.
12 Carefully decant and discard the supernatant without disturbing the cellular pellet.
  Rationale: Centrifugation concentrates cellular material into a pellet while removing excess fluid.

#### Cell block preparation

13 To the cellular pellet, add 5 drops of plasma and 5 drops of Innovin reagent (1:1), which was the standardized volume used for cell block preparation. If <5 drops of plasma are available, add an equal volume of Innovin reagent to maintain a 1:1 ratio. Mix gently and allow the mixture to stand for 10 min at room temperature to promote clot formation.
14 Centrifuge for 5 min at 725 × *g*, room temperature.
15 Recover the resulting coagulated cellular material for routine cell block processing and paraffin embedding.
  Expected outcome: Formation of a firm cellular clot suitable for histological sectioning, immunohistochemistry, and molecular testing.

#### ThinPrep cytology processing

16 To the cellular pellet obtained after the first centrifugation (step 11), add:
  2 ml glacial acetic acid
  2 ml CytoLyt solution.
17 Mix thoroughly and incubate for 10 min at room temperature.
18 Centrifuge for 5 min at 725 × *g*, room temperature.
19 Decant the supernatant carefully.
20 Resuspend the remaining cellular pellet and transfer it to the original ThinPrep vial containing CytoLyt solution.
21 Load the vial into the ThinPrep 5000 Processor according to the manufacturer's instructions.
22 Allow the instrument to prepare a standardized monolayer cellular deposit on a glass slide.
  Rationale: Acetic acid and CytoLyt reduce blood, mucus, and proteinaceous debris, improving microscopic visualization.

#### Anticipated results

Properly processed specimens should yield:

(1) Uniform cellular distribution.
(2) Minimal background blood and debris.
(3) Preserved cellular morphology.
(4) Adequate material for cytological diagnosis.
(5) Additional material available for cell block preparation, immunohistochemistry, and molecular analyses.

## ALTERNATIVE ThinPrep FNA PROCESSING USING CYTOLYT AND PRESERVCYT

This alternative approach follows the workflow described in the ThinPrep FNA Quick Reference Guide provided by Hologic (see Internet Resources).

### Additional Materials (also see Basic Protocol)

Vortex mixer

1 As an alternative to direct collection in PreservCyt, collect thyroid FNA specimens directly into 30 ml of CytoLyt solution.
  If possible, flush the needle and syringe with a sterile anticoagulant solution before aspirating the sample. Some anticoagulants may interfere with other cell processing techniques, so use caution if you plan to use the specimen for other testing.
2 Concentrate by centrifuging 10 min at 600 × *g*, room temperature, or 5 min at 1200 × *g*, room temperature.
3 After centrifugation, completely remove the supernatant and resuspende the cellular pellet by vortexing or gentle pipetting.
4 Evaluate the appearance and size of the cellular pellet and transfer an appropriate amount of the resuspended specimen to a fresh PreservCyt vial according to the pellet size as follows:
  a. If the pellet is clearly visible and <1 ml, place the centrifuge tube in a vortex or resuspend the cells in the residual liquid or mix the pellet by syringing it manually with a pipette. Transfer 2 drops of the pellet to a fresh PreservCyt solution vial.
  b. If the pellet is not visible or is scant, add the contents of a fresh PreservCyt solution vial (20 ml) into the tube. Vortex briefly to mix the solution and pour the entire sample back into the PreservCyt solution vial.
  c. If the pellet is >1 ml, add 1 ml of CytoLyt solution into the tube. Vortex briefly to resuspend the pellet. Transfer 1 drop of the specimen to a fresh PreservCyt solution vial.
5 Allow to stand in PreservCyt for 15 min before processing on the ThinPrep system.
6 Load the vial into the ThinPrep 5000 Processor according to the manufacturer's instructions
  This alternative approach follows the workflow described in the ThinPrep FNA Quick Reference Guide provided by Hologic.

## REAGENTS AND SOLUTIONS

### CytoLyt solution

Use CytoLyt commercial methanol‐based cell wash solution (Hologic, cat. no. 70408‐002) directly without further preparation. Store at room temperature until the manufacturer's expiration date. Mix gently before use if stored for prolonged periods.

### Ethanol, 96%

Use 96% ethanol, analytical grade (Sigma‐Aldrich or equivalent) directly without dilution. Store at 2° to 30°C according to the manufacturer's expiration or retest date.

### Glacial acetic acid

Add 2 ml glacial acetic acid (ACS reagent grade; Sigma‐Aldrich, cat. no. A6283 or equivalent) directly to the cellular pellet immediately before adding CytoLyt solution (see recipe). Mix thoroughly. Prepare immediately before use.

### Human plasma solution

Thaw commercial human plasma suitable for laboratory use (or validated institutional plasma) completely before use and mix gently before dispensing. Store frozen according to the manufacturer's recommendations. Once thawed, store at 4°C and use within the manufacturer's recommended stability period.

### Innovin reagent

Prepare Innovin thromboplastin reagent (Siemens Healthineers, cat. no. B4212‐50) according to the manufacturer's instructions. Store at 2° to 8°C for up to 10 days after reconstitution in the closed original vial. Bring to room temperature before use. Do not freeze.

### PreservCyt solution

Use PreservCyt solution collection vial containing 20 ml commercial methanol‐based preservative solution (Hologic, cat. no. 70787‐002) directly without further preparation. Store at room temperature protected from excessive heat and direct sunlight. Follow the manufacturer's expiration date.

## COMMENTARY

### Critical Parameters

Perform the following quality control tasks while executing the Basic Protocol:

(1) Verify patient identification at both specimen reception and processing stages.
(2) Ensure adequate fixation and preservation conditions.
(3) Confirm formation of an adequate cellular pellet after centrifugation.
(4) Document any limitations, including scant cellularity or excessive blood contamination.
(5) Review prepared slides for cellular adequacy before final interpretation.

### Troubleshooting

Common technical issues that may be encountered during the processing of thyroid FNA specimens using the ThinPrep liquid‐based cytology system, together with their possible causes and recommended corrective actions, are summarized in Table 1. This troubleshooting guide is intended to assist laboratory personnel in identifying and resolving procedural problems that may affect specimen adequacy, slide quality, and the reliability of cytological interpretation.

**Table 1:** Common Technical Problems, Possible Causes, and Recommended Solutions During ThinPrep Processing of Thyroid Fine‐Needle Aspiration Samples

Problem	Possible Cause	Solution
Low cellularity specimen	Cellular loss during centrifugation or decantation	Thoroughly rinse the needle and syringe in PreservCyt and avoid loss of cellular sediment during decantation
Absence of cellular sediment after centrifugation	Centrifuge imbalance, insufficient centrifugation speed, or inadequate centrifugation time	Verify centrifugation parameters or perform a second centrifugation step
Excessive blood in the slide	Insufficient hemolytic effect of CytoLyt and acetic acid treatment	Consider repeating the hemolytic treatment
Insufficient or incomplete cellular deposition on the ThinPrep slide	Partial filter obstruction, low cellular concentration, or excessive mucus	Verifying instrument maintenance and performing pre‐treatment with CytoLyt
Thick or uneven cellular deposition on the slide	Excessive mucus or proteinaceous material	Dilute the sample with additional CytoLyt and repeat processing
Altered cellular details	Prolonged storage or delayed processing	Process the specimen as soon as possible after collection
Failure of clot formation for cell block preparation	Inadequate plasma quality, inappropriate proportions, or low cellularity	Verify reagents and increase plasma volume or coagulation time
ThinPrep filter obstruction	Excessive mucus, blood, or proteinaceous material	Perform pre‐treatment with acetic acid and CytoLyt or filter the sample beforehand
Cross‐contamination between specimens	Inadequate equipment cleaning or identification errors	Apply strict traceability procedures and cleaning protocols between specimens

### Understanding Results

Successful implementation of this protocol should yield high‐quality ThinPrep cytology preparations characterized by well‐preserved thyroid follicular cells distributed within a relatively uniform monolayer and a clean background with minimal obscuring material. Appropriate specimen processing reduces excess blood, mucus, and proteinaceous material while preserving cellular morphology, thereby facilitating accurate cytomorphological evaluation according to The Bethesda System for Reporting Thyroid Cytopathology (TBSRTC).

Adequately processed ThinPrep slides should demonstrate well‐preserved nuclear and cytoplasmic features, limited cellular overlap, and a background that allows clear assessment of diagnostically relevant features. Depending on the characteristics of the specimen and the underlying thyroid lesion, these may include cellularity and architectural patterns, nuclear enlargement, chromatin characteristics, nuclear grooves, intranuclear pseudoinclusions, colloid, inflammatory cells, and other background elements. Cellular preservation should be sufficient to support reliable cytomorphological interpretation and classification within the appropriate Bethesda diagnostic category.

The amount and distribution of cellular material may vary among specimens. Some preparations may show a relatively uniform and cellular monolayer, whereas others may be sparsely cellular or contain three‐dimensional follicular cell groups and variable amounts of colloid. These variations should be interpreted in the context of specimen adequacy and the cytomorphological criteria applicable to the specific diagnostic category. Representative examples of ThinPrep preparations demonstrating different cellular patterns and their corresponding cell block preparations are shown in Figure 1 and Figure 2.

**Figure 1:**
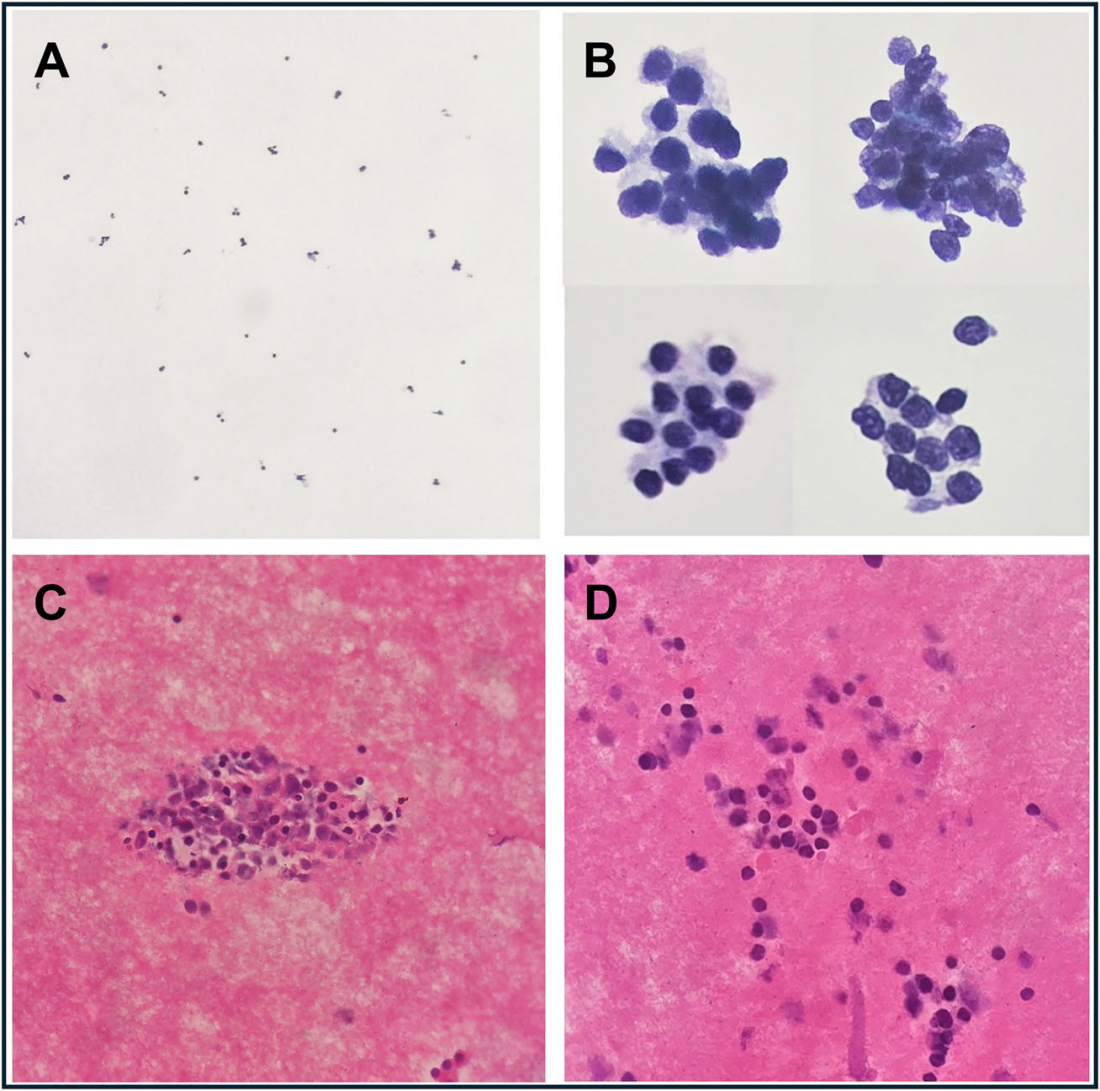
Representative ThinPrep cytology and corresponding cell block preparations demonstrating adequate specimen processing and cellular preservation. (**A**) ThinPrep liquid‐based cytology preparation (Papanicolaou stain, 4× magnification) showing a relatively uniform distribution of cellular material on a clean background, with minimal obscuring blood and processing artifacts. (**B**) ThinPrep liquid‐based cytology preparation (Papanicolaou stain, 40× magnification) demonstrating adequate preservation of follicular cell cytomorphology and nuclear detail. (**C**) Histological section of a cell block prepared from residual material preserved in a PreservCyt vial (hematoxylin and eosin stain, 10× magnification), demonstrating successful recovery of cellular material suitable for paraffin processing. (**D**) Histological section of the corresponding cell block (hematoxylin and eosin stain, 10× magnification), showing preserved cellular morphology and cellular material potentially suitable for ancillary studies, depending on specimen cellularity and clinical requirements.

**Figure 2:**
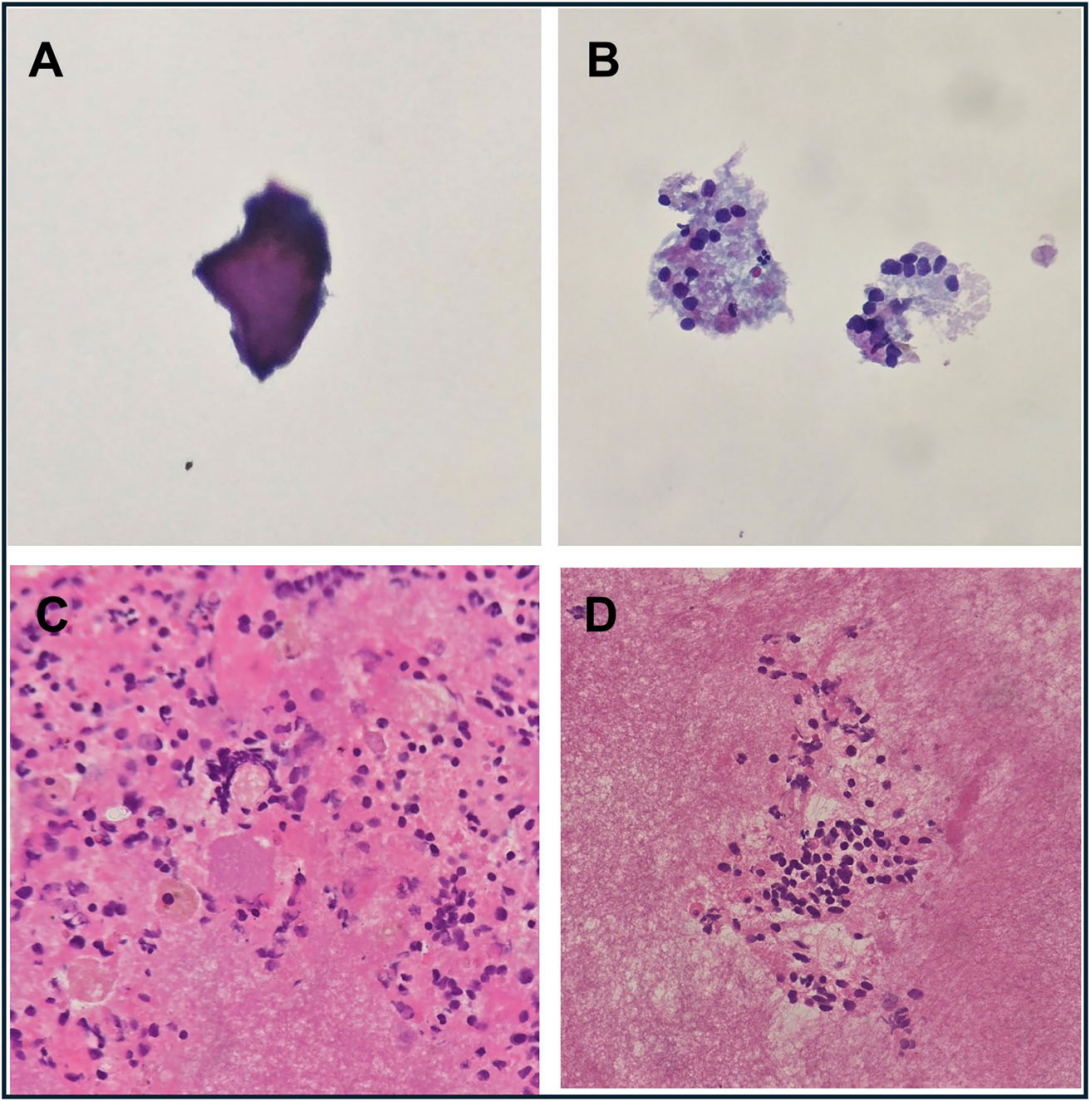
Representative ThinPrep cytology and corresponding cell block preparations showing variable cellularity, follicular cell groups, and colloid. (**A**) ThinPrep liquid‐based cytology preparation (Papanicolaou stain, 40× magnification) showing a sparsely cellular preparation with colloid material and scattered cellular elements on a clean background with minimal obscuring hemorrhage. (**B**) ThinPrep liquid‐based cytology preparation (Papanicolaou stain, 40× magnification) demonstrating three‐dimensional groups of follicular cells with preserved cytomorphological and nuclear features and scant background colloid. (**C**) Histological section of a cell block prepared from residual material preserved in a PreservCyt vial (hematoxylin and eosin stain, 10× magnification), demonstrating recovery of cellular and colloid material suitable for paraffin processing, with preservation of cellular architecture. (**D**) Histological section of the corresponding cell block (hematoxylin and eosin stain, 10× magnification), showing morphologically preserved groups of follicular cells associated with colloid material. When sufficient cellularity is present, the cell block may be suitable for ancillary studies, including immunohistochemistry and molecular testing.

Successful cell block preparation should yield recoverable cellular material suitable for routine histological processing and paraffin embedding. Depending on the cellularity of the residual PreservCyt material, histological sections may demonstrate cohesive groups of follicular cells, colloid, and/or preserved tissue fragments. When sufficient material is available, the cell block may also provide a substrate for ancillary studies, including immunohistochemistry and molecular analyses, such as DNA‐ and RNA‐based testing, when clinically indicated.

### Time Considerations

The complete processing of thyroid fine‐needle aspiration (FNA) specimens using the ThinPrep liquid‐based cytology system can be completed within 30 to 45 min per specimen, excluding specimen collection and microscopic evaluation. Specimen fixation in CytoLyt solution requires 15 to 30 min to ensure adequate preservation of cellular morphology. Centrifugation and specimen preparation generally require 10 to 15 min, followed by 2 to 3 min for automated slide preparation using the ThinPrep processor. Slide fixation and staining (e.g., Papanicolaou stain) typically require an additional 20 to 30 min, depending on the laboratory workflow and staining platform. Once staining is completed, cover slipping and slide drying require 5 to 10 min before the slides are ready for microscopic examination by the cytopathologist.

In laboratories processing multiple specimens, the automated ThinPrep system allows batch processing, thereby reducing hands‐on time and improving workflow efficiency.

### Author Contributions

**Vargas‐Castellanos Elizabeth**: Conceptualization; writing—original draft; writing—review and editing. **Arenas Avila Lina Alejandra**: Methodology; validation. **Rocha Franky**: Conceptualization; writing—review and editing.

### Conflict of Interest

The authors declare that they have no competing financial interests. The ThinPrep system and related reagents are commercially available products; however, the manufacturer had no role in the design of the protocol, data interpretation, manuscript preparation, or the decision to publish.

## Data Availability

Data sharing not applicable to this article as no datasets were generated or analyzed during the current study.
